# Selections

**Published:** 1856-09

**Authors:** 


### SELECTIONS.

*On Carbonic Acid Gas, as a Local Anæsthetic in Uterine Diseases, &c.*

At a regular meeting of the Academy of Medicine, of New York, March 5th, 1856, Dr. Willard Parker, President, a communication was read from Prof. Simpson, of Edinburgh, addressed to the Academy.

It appears that Prof. Simpson was led to the use of carbonic acid gas as a local anæsthetic in painful conditions of the vagina, uterus, and neighboring parts, from reading the case of a lady, treated by Dr. Rossi, of Italy, and reported in the second edition of *Pereira's Materia Medica*, Vol. I, p. 155. In this case there was no organic disease, and merely an increased irritability, which was completely relieved by the injection of carbonic acid gas.

Prof. Simpson has frequently resorted to this treatment within the last two or three years; and, though not always with success, yet frequently with great relief, and occasionally with immense benefit. Several cases were given in illustration. One lady who had been bed-ridden for years from pain and bearing down when standing erect, was almost entirely cured by injections of this gas.

His method of applying it, is, to use a bottle having a flexible tube attached to the cork. The materials used for generating the gas, are tartaric acid, ʒvi.; bi-carbonate of soda in solution, ʒviii.; and water, ℥vi. The injection may be used several times a day. Other materials may be used.

Prof. Simpson adds, that the employment of carbonic acid gas as a local anæsthetic to the uterine mucous surface and other parts of the body, is not a recent discovery. Dr. Dewees, of Philadelphia, speaks favorably of it in his work (*Dis. of Fem.*, p. 269). Prof. Mojon, of Geneva, has used it frequently and with decided advantage.

Referring to ancient writers, the author is disposed to consider the practice of burning certain aromatic and medicinal herbs, and applying the fumes to the interior of the vagina by means of proper tubes, to be but another phase of this practice —as cabonic acid is the result of such combustion.

He also ascribes the beneficial effects of mineral waters, in many cases at least, to the topical application, by means of baths and injections, of these waters, which generally hold in solution large quantities of carbonic acid. Female patients have assured him of the relief they experienced from uterine pains while using injections of the waters of springs, as practiced at different German baths. The same is true in certain cutaneous diseases.. The common effervescing draught, in gastric irritability and nausea, acts on the same principle. The injection of carbonic acid gas in dysentery, as practiced with success by Hey, of Leeds, in 1772, Perkins, etc., is directly in point. The benefit of the common yeast poultice, which gives rise to carbonic acid gas, may be similarly explained. Many other examples were alluded to in the paper, showing how frequently this agent is used in practice without recognition of its anodyne properties.

Dr. Detmold remarked, that members would recollect, that, about the year 1847, he called the attention of the Academy to certain propositions which he then made, proving quite conclusively that carbonic acid gas is the efficient agent in causing anæsthesia. The carbonic acid may be given as such, or one of its chemical ingredients may be so administered, that, finding in the blood the other constituents of this compound, carbonic acid gas is generated, and anæsthesia to a certain extent is the result. Thus, we may administer oxygen in large quantities, in the form of nitrous oxyde, (protoxide of nitrogen or laughing gas,) which has all the chemical reactions of oxygen, but is much more soluble in water and the serum of the blood than pure oxygen, and, therefore, is much more readily taken up. This compound meeting with the carbon of the blood, carbonic acid gas is formed in large quantities, with the production of anæsthesia to a certain extent. Or we may, on the contrary, administer the carbon, as the oxide of carbon or any of the hydro-carbons, alcohol, the ethers, etc.; in this case the blood again furnishes the other constituent of carbonic acid, (oxygen,) and anæsthesia is again the result.

The stage of excitement corresponds to the period of combination of these elements and the formation of carbonic acid gas. If the gas is administered as such, there will be no stage of excitement, but if the constituents combine slowly, and the gas is generated in limited quantities, there will be a corresponding stage of excitement. Thus, in the stupor of drunkenness, carbonic acid is exhaled in normal quantities, but as the stupor passes off large quantities of that gas are exaled. The venous state of the arterial blood during anæsthesia, is another proof that carbonic acid is being generated in large quantities. If it is true that in post mortem examinations of those dying while under the influence of chlorform, bubbles of air are found in the heart and blood vessels, it is highly probable that this air is carbonic acid gas, unless, perchance, it has entered the circulation by some mechanical lesion.

The only means, in his opinion, of any avail in restoring a patient from profound or fatal anæsthesia, is artificial respiration, or such other means as, by exciting reflex action, will restore respiration, and thus hasten the elimination of the carbonic acid gas. It has been recommended, in threatened and apparent death from anæsthesia, to resort to the inhalation of oxygen or nitrous oxide. Reasoning from the premises which he had given, such remedies would be in the highest degree dangerous. To satisfy himself in regard to this fact, he had made numerous experiments upon animals, and invariably found a fatal issue hastened by administering oxygen.—*N. Y. Jour. of Medicine.*

At a subsequent meeting, Dr. Detmold favored the Academy with a written exposition of his views of the rationale of the action of chloroform, sulph. ether, and nitrous oxide, the three agents employed for the purpose of producing anæsthesia. He attributes the action of all of them to the production of carbonic acid gas *in the system.* The first two supply the carbon, which absorbing oxygen from the blood, and the last supplying oxygen, which absorbing carbon, in either case carbonic acid is the result, which by its action on the living organism produces anæsthesia. This theory, though not absolutely susceptible of demonstration, is yet apparently based on a logical foundation, and finds a seeming confirmation in a number of well-known facts; indeed, it was elicited by the allusions made to the anæsthetic properties of carbonic acid, by Prof. Simpson in his recent paper, of which I gave an account in my previous letter. —*Med. and Surg. Reporter.*

---

*The Treatment of Ague by Iodide of Potassium.* By E. F. SANKEY.

Ever since I have resided in this village, (now for five years,) I have been dissatisfied with the usual treatment of ague by quinine, as in some cases the disease yielded to that remedy, and in others did not. But I could think of no other treatment likely to be successful, though I tried many, including arsenic, till, some three years ago, I read in a number of the *Medico-Chirurgical Review* (I forget which) that the German pathologists considered that the congested spleen (ague cake) was the cause and not the effect of the disease; and I remembered that Dr. Williams had written on the efficacy of bromide of potassinm in such a lesion; but not having that drug in my surgery, I determined to try the iodide of potassium instead in the next case of ague that came before me, intending, if that failed, to procure the bromide. But I am happy to say that the object of my writing is to state to my professional brethren that I have used the iodide of potassium now in considerably more than a hundred cases, and have never yet failed in curing the disease very quickly. In some cases, where the disease has been of long standing, and the patient very much reduced, I have added a grain or two of quinine to each dose of the iodide of potassium; but my general prescription has been for an adult: ℞.—Potas. iodid., ʒiss.; aquæ menth. pip., ℥xj. M. Coch. Mag., ij. 4ta quaque hora sumend. So that there could be no doubt what was the remedy that cured the disease. In proof of the value of this drug, I will only mention one case out of all that I have thus treated.

Mrs. Smith sent for me early last month, having suffered from tertian ague, off and on, since September. Not being in very good circumstances, she went to the clergyman's wife of the parish in which she resided, who very kindly gave her some quinine, telling her it was no use sending for the medical man, as he *must* give her the same remedy. However, not getting well, she sent for me. After hearing what I have related, and finding she had a tolerable pulse, her bowels open and motions healthy, with a clean tongue. I sent her nothing but the above mixture; and she never had a return of the ague after the second she took of it.

I shall be glad if, by inserting this letter, other medical men will try this remedy, and report to you their experience.—*Assoc. Med. Jour.*

---

*The Influence of Occupation on Mortality.*

The attempts hitherto made to determine the influence of professions on health are greatly reduced in value, in consequence of the inadequate data on which they are based. If Ramazzini and Thackrah could have known the facts of the last census, the observations resulting from their scientific and benevolent labors would have had the authority of natural law. That census sheds the light of statistical truth on the relation of professions and occupations to mortality, and brings out truths which no less extensive investigation would enable the most sagacious observer to anticipate.

It will be an incalculable advantage to obtain, by means of the next census, a scientific deduction as to the effect of each kind of occupation on mortality. As the initial step has been taken, we may expect it will be followed by others, in a path which, if beset with difficulties, cannot be traversed without leading to the most important and beneficial discoveries.

The last Census Report gave the number of persons in each occupation in 1851, and the Fourteenth Annual Report of the Registrar-General shows the numbers in those occupations dying at corresponding ages. In this early attempt to arrive at the ratio of occupation to the rate of mortality, it has been found that a difficulty arises from the want of definition of the various occupations, sufficiently clear and determined—a difficulty which can be overcome by giving more detail to future census operations. It is, for example, found impossible to distinguish the rate of mortality among the different classes engaged in the manufacture of silk, of cotton, of linen, and of woolen, as great numbers of them are grouped together under the designation of "Weavers." "Miners," whether in lead, iron, copper, or coal mines, fall under one general designation; and "Laborers," in the field or railways, in quarries and on the roads, are not distinguished from each other in the registers.

Still, there are certain occupations sufficiently defined to obviate all danger of their being confounded, and whose rate of mortality can now be recorded with certainty. We give these classes as the decennial period, ranging from 45 to 55,* as arranged in a Table (XVII) which shows the advancing rate of mortality in twelve occupations.

1. *Farmers.*—Of the twelve classes under consideration, farmers are the longest livers, their rate of mortality being not quite 12 in 1000 (11.99). The number of English farmers of all ages, 1851, including 2,429 graziers, was 225,747, of whom there were 53,608 between the age of 45 and 55. In that year, the total number of deaths among farmers of all ages, was 6,426, very much below the numbers which would have been registered had these individuals been engaged in other pursuits. These facts prove that the pure air, the daily exercise, the substantial fare, and the other aids to health enjoyed by this substantial class, considerably modify the influence of unfavorable weather, bad seasons, open ports, peculiar burdens on land, and all the other ruinous things which farmers' friends have been accustomed to depict in such gloomy colors.

2. *Shoemakers* hold the next place to farmers, their rate of mortality between 45 and 55 being 15.3 in 1000. They are followed by—

| | | |
|---|---|---|
| 3. *Weavers*, | 15.37 | in 1000. |
| 4. *Grocers*, | 15.79 | " |
| 5. *Blacksmiths*, | 16.51 | " |
| 6. *Carpenters*, | 16.67 | " |
| 7. *Tailors*, | 16.74 | " |
| 8. *Laborers*, | 17.30 | " |

As will be seen, on inspection, there is among these seven occupations a gradual increase in the rate of mortality, which, considering their great diversity, is quite remarkable. The near approach of these occupations to each other in the scale of mortality, arises from the circumstance that they have peculiar dangers which tend to counterbalance each other. Thus it is to be noticed that the "tailor is not exposed to the explosions which are fatal to the miner, and the laborer has exercise which is denied to the tailor."

Ascending this scale of danger, we pass to—

| | | |
|---|---|---|
| 9. *Miners*, | 20.15 | in 1000. |
| 10. *Bakers*, | 21.21 | " |
| 11. *Butchers*, | 23.10 | " |
| 12. *Innkeepers*, | 28.34 | " |

A great disparity is observable in passing from laborers into

* The decade from 45 to 55 is the only age to which the Census Returns have been applied in the Fourteenth Annual Report of the Registrar-General. We shall have still more important results when these returns are applied to earlier ages.

the class of miners, telling a tale of dangers, many of which result from criminal neglect. Between laborers and the last four classes in this table there is a remarkable hiatus. In the classes previously noticed, the difference in no case is no more than one in a thousand, and in some instances less. Here the difference begins with three, and mounts up to nine, in a thousand.

The returns show that the highest rates of mortality are found among the butchers (23.10 in 1000), and the class of innkeepers and licensed victuallers (28.34 in 1000).

The extraordinary mortality of butchers is a fact for which we are indebted wholly to the last census. The "red-injected face" of the butcher has produced a wrong idea as to the healthy nature of his occupation. This idea is now corrected by scientific induction, and proper sanitary means will overcome the evil thus brought to light. To quote the significant remarks in the report conveying this fact, here is an important problem for solution:—"On what does the great mortality of the butcher depend? On his diet, into which too much animal food and too little fruit and vegetables enter? on his drinking excess? on his exposure to heat and cold? or, which is probably the most powerful cause, on the elements of decaying matter by which he is surrounded in his slaughter-house and its vicinity?"

If the rate of mortality among innkeepers, licensed victuallers, and beer-shop keepers should be siezed with avidity by the advocates of teetotalism, they must not be forbidden its use; at the same time they must be reminded, that "many highly-respectable men of this class lead regular lives, and are of steady habits; but others, exposed by their business to unusual temptations, live intemperately and enjoy less quiet at night than the rest of the community. They are exposed also to zymotic diseases by intercourse with large numbers of people."

Startling and painful as are these disclosures, they cannot be too widely published. They have a practical value among those who deal with the averages of life for commercial or benevolent purposes; while, to those more specially concerned, they show the necessity, for their own safety, of employing the measures by which unnecessary disease and premature death may be obviated—*Med. Times and Gazette.*

